# Matrix stiffness regulates NPC invasiveness by modulating a mechanoresponsive TRPV4-Nox4-IL-8 signaling axis

**DOI:** 10.7150/jca.104235

**Published:** 2025-01-13

**Authors:** Peng Zhang, Dunhui Yang, Kang Li, Jin Zhang, Zhen Wang, Fang Ma, Xianqin Liao, Shibo Ma, Xianhai Zeng, Xiangmin Zhang

**Affiliations:** 1 Department of Otolaryngology, Longgang Otolaryngology hospital & Shenzhen Key Laboratory of Otolaryngology, Shenzhen Institute of Otolaryngology, Shenzhen, Guangdong, China; 2 Department of Otolaryngology, The Second People's Hospital of Yibin, Yibin, Sichuan, China

**Keywords:** Matrix stiffness, TRPV4, NOX4, tumorigenesis, NPC

## Abstract

Matrix stiffness is a critical determinant of tumorigenesis and cancer progression. Transient receptor potential vanilloid 4 (TRPV4), a mechanosensitive calcium channel, regulates angiogenesis and stromal stiffness in various tumors. However, it is unclear whether matrix stiffness regulates the invasiveness of nasopharyngeal carcinoma (NPC) cells through TRPV4. In this study, we found that increased matrix stiffness of NPC tissues correlated with advanced tumor stages. Furthermore, simulation of high matrix stiffness *in vitro* upregulated TRPV4, and increased the migration, invasion, and epithelial mesenchymal transition (EMT) of NPC cells. Knockdown or pharmacological inhibition of TRPV4 significantly suppressed the calcium influx in NPC cells, and inhibited their invasiveness and EMT under high-stiffness conditions. Mechanistically, TRPV4 modulated the invasiveness of NPC cells in response to matrix stiffness via the NOX4/IL-8 axis. Notably, TRPV4 and IL-8 levels were significantly increased in the high-stiffness NPC tissues, and showed a positive correlation. Taken together, matrix stiffness promotes the malignant progression of NPC cells through the activation of the TRPV4/NOX4/IL-8 axis, which could be explored further as a potential target for NPC therapy.

## Introduction

Nasopharyngeal carcinomas (NPCs) are epithelial tumors that originate from the mucosal tissues in the nasopharynx 1, particularly the anterior parietal wall and the pharyngeal crypts in the lateral wall. The incidence of NPC is especially higher in southern China and Southeast Asia 2, and the risk factors include genetic susceptibility, EBV infection, environmental triggers and dietary habits 3. At present, the clinical management of NPC relies on radiotherapy, chemotherapy and surgery 2. However, due to their obscure anatomical location, NPCs are often difficult to detect at the early stages, and many patients are diagnosed at the middle or late stage of the disease, leading to poor prognosis. Therefore, it is necessary to explore the mechanisms underlying NPC development and identify new diagnostic markers and therapeutic targets.

**M**atrix stiffness is a characteristic feature of the tumor microenvironment (TME). Under normal physiological conditions, the extracellular matrix (ECM) has a certain degree of stiffness to maintain the normal structure and function of tissues. However, matrix stiffness increases significantly during tumor formation and progression, which allows the tumor cells to proliferate, migrate and invade. Furthermore, changes in the ECM can activate the pro-oncogenic genes and pathways, thus promoting tumor progression. For instance, the cancer-associated fibroblasts (CAFs) in oral tumors increase matrix stiffness by producing LOX, which in turn promotes cancer progression through the FAK phosphorylation pathway 4. In hepatocellular carcinoma (HCC) as well, increased stromal stiffness is known to promote neo-angiogenesis 5. Furthermore, high matrix stiffness in the NPC environment accelerates cell migration, invasion, and the formation of invasive pseudopods, and is closely related to prognosis 6. The NPC stroma becomes progressively denser and stiffer as the tumor grows, which has a significant impact on the efficacy of radiation therapy 7. However, the mechanisms underlying matrix stiffness in NPC have not been fully clarified.

Matrix stiffness can induce intracellular receptors or sensors, such as TRPV4, that modulate cellular behavior. Knocking down TRPV4 in human epidermal keratinocytes has been shown to prevent excessive stiffening of the ECM 8, 9. TRPV4 is a calcium (Ca^2+^) permeable non-selective cation channel 10 that controls angiogenesis by acting on the tumor endothelial cells, and is associated with matrix stiffness. It is activated by various physical factors, including temperature, pressure, mechanical forces, osmolality, and ultraviolet light 11. Recent studies have shown that TRPV4 is aberrantly expressed in cancers 12. In a previous study, we had shown that TRPV4 promotes NPC progression through NFAT4 signaling 13. However, TRPV4 activation has not been investigated in the response of NPC cells to matrix stiffness, and the underlying mechanisms are still unknown. High levels of reactive oxygen species (ROS) play a crucial role in tumor formation, progression, and metastasis. For instance, ROS production significantly affects the proliferation and migration ability of pancreatic cancer cells, and mediates the progression of NPC through AKT/mTOR signaling 14. TRPV4 activation enhances mitochondrial ROS production in response to cellular stress due to increased calcium influx 15. ROS is primarily generated during oxidative phosphorylation by the NADPH oxidase (NOX) family and the mitochondrial electron transport chain. High NOX expression is associated with increased ROS production, leading to oxidative stress. We had previously shown that up-regulation of TRPV4 in obese mice enhanced NOX-mediated ROS generation and increased vascular permeability 16. However, it is unclear whether the TRPV4/NOX axis is involved in matrix stiffness-dependent progression of NPC.

In this study, we observed higher matrix stiffness in the advanced stage NPC tissues, which correlated with increased TRPV4 expression and metastasis. Furthermore, downregulation or pharmacological inhibition of TRPV4 significantly inhibited matrix stiffness-induced migration, invasion, and epithelial-mesenchymal transition (EMT) of NPC cells through the NOX4/IL-8 axis. Overall, our study indicates that matrix stiffness regulates the aggressiveness of NPC tumors via the TRPV4/NOX4/IL-8 axis.

## Methods and Materials

### Drugs

GSK1016790A (HY-19608), HC-067047 (HY-100208), apocynin (HY-N0088) and GLX351322 (HY-100111) were purchased from MedChemExpress. Recombinant human IL-8 was from Sigma-Aldrich. The anti-TRPV4 antibody (ACC-034) was purchased from Alomone Labs, and anti-IL-8 antibody was purchased from Proteintech. DAPI was purchased from Beyotime. Fluor-488 donkey anti-rabbit IgG, Fluo-4/AM and CMH2DCFDA were purchased from Thermo Fisher.

### Ethics statement

The experiments involving clinical samples were approved by the Review Board of Longgang Otolaryngology hospital, and the experiments conformed to the principles contained in the World Medical Association Declaration of Helsinki. Informed consent was requested and obtained from all participants for collecting anonymous specimens.

### Cell culture

The human NPC cell lines 5-8F and CNE1 have been described previously 14. The cells were cultured in DMEM or RPMI1640 containing 10% fetal bovine serum (FBS), 100 U/ml penicillin and 100 μg/ml streptomycin at 37 °C under 5% CO_2_. To simulate the tumor stroma *in vitro*, the cells were cultured in hydrogel-coated wells (Matrigen, Irvine, CA, USA) at 0.2 kPa (low stiffness) and 25 kPa (high stiffness).

### Quantitative PCR (qPCR)

Total RNA was extracted using the RNeasy Mini kit (Qiagen) according to the manufacturer's instructions, and reverse transcribed using the RT Master Mix for qPCR kit (MedChemExpress). QPCR was performed using SYBR Green qPCR Master Mix (MedChemExpress) on the Applied Biosystems 7500 FAST Real Time PCR System 14. The primer sequences were shown in Supplementary Table S1.

### Measurement of intracellular Ca^2+^ levels

The NPC cells were stimulated with GSK1016790A, and then treated with 0.02% Pluronic F-127 (Invitrogen) and 10μM Fluo-4/AM (Invitrogen) for 30 min in the dark. The fluorescence intensity relative to the baseline (F1/F0) was calculated to quantify the changes in cytoplasmic Ca^2+^ levels 13.

### ROS staining

Intracellular ROS levels were measured using CMH2DCFDA (Molecular Probes) as previously described 14. Briefly, cells were seeded in confocal dishes and incubated with CMH2DCFDA for 15 min at 37 °C, followed by Hoechst 33342 staining for 10 min. Images were taken with a Leica TCS SP5 confocal microscope.

### siRNA transfection

All siRNAs were cloned into the lentiviral transfer vector psi-LVRU6GP and virus particles were obtained from GeneCopoeia. The siRNA sequences were shown in Supplementary Table S2.

### Immunohistochemistry

Immunohistochemistry was performed as previously described 17. Each specimen was scored according to the intensity of staining (0, none; 1, weak; 2, moderate; 3, strong) and the proportion of stained cells (0, 0%; 1, 1-24%; 2, 25-49%; 3, 50-74%; 4, 75-100%) according to the German semi-quantitative scoring system. The final immunoreactivity score was determined by multiplying the intensity with the positivity rate, and ranged from 0 to 12.

### Masson's trichrome staining

The collagen fibers in NPC specimens were stained with Masson's trichrome solution (Solarbio) as previously described 17. Matrix stiffness was quantified by calculating the percentage of collagen fibers (stained blue) in the tumor stroma as follows: 0 = <10%, 1 = 11-30%, 2 = 31-50%, 3 = 51-80%, 4 = >80%. Based on the scores, the specimens were classified as “low-stiffness” (score 0 to 2) and “high-stiffness” (score 3 to 4).

### Immunofluorescence staining

Immunofluorescence staining was performed as previously described. The cells were incubated with anti-TRPV4 antibody at 4 °C, followed by Alexa Fluor-488 donkey anti-rabbit IgG (1:500) (Invitrogen) for 1h at room temperature. After counterstaining with DAPI (Beyotime), the cells were imaged with a Leica TCS SP5 confocal microscope.

### Migration and invasion assays

Cell migration and invasion assays were performed using transwell chambers (Corning) as previously described 18. Briefly, cells were seeded into the upper chamber in serum-free medium. The medium containing 10% FBS was placed in the lower chamber and the cells were further incubated for 24 h, cells in the upper chamber were removed with a cotton swab, and the rest of the membrane had invaded the cells. Cells migrated through the membrane were fixed with 4% paraformaldehyde and stained with crystal violet. Cell invasion was determined with Matrigel matrix (BD Biosciences) coated on the upper surface of the transwell chamber. Cells were seeded, fixed and stained as described in migration assay.

### Cell adhesion assay

The suitably treated cells were incubated in 24-well plates for 1h. The floating cells were removed and counted, and the adherent cells were detached using trypsin and then counted. The percentage of adherent cells was calculated 19.

### Cell detachment assay

The NPC cells were incubated in 24-well plates for 24 h, and then digested with 0.05% trypsin for 3 minutes. The harvested cells were counted, and the percentage of detached cells was calculated 19.

### ELISA

The concentration of IL-8 in the supernatants of cultured 5-8F and CNE1 cells was measured using the Human IL-8 ELISA Kit (Proteintech) according to the manufacturer's instructions. Briefly, standards were diluted, and samples were added to wells and incubated; the wells were washed, after which antibodies were added. After washing, colour development reagents were added. The absorbance (OD) value of each well was measured sequentially at a wavelength of 450 nm. The measurement was performed within 15 min after the addition of stop solution. The corresponding concentration from a standard curve was multiplied by the dilution factor to calculate the concentrations of IL-8.

### Statistical analysis

Two-tailed Student's t-test was used to compare the data of two groups, and one-way ANOVA was used for analyzing multiple groups. All analyses were conducted using GraphPad Prism software version 5.0. The data were expressed as the mean ± standard error (SEM) of at least three independent experiments. P value less than 0.05 was considered statistically significant.

## Results

### Matrix stiffness correlates with advanced stages and TRPV4 expression

**Figure 1:**
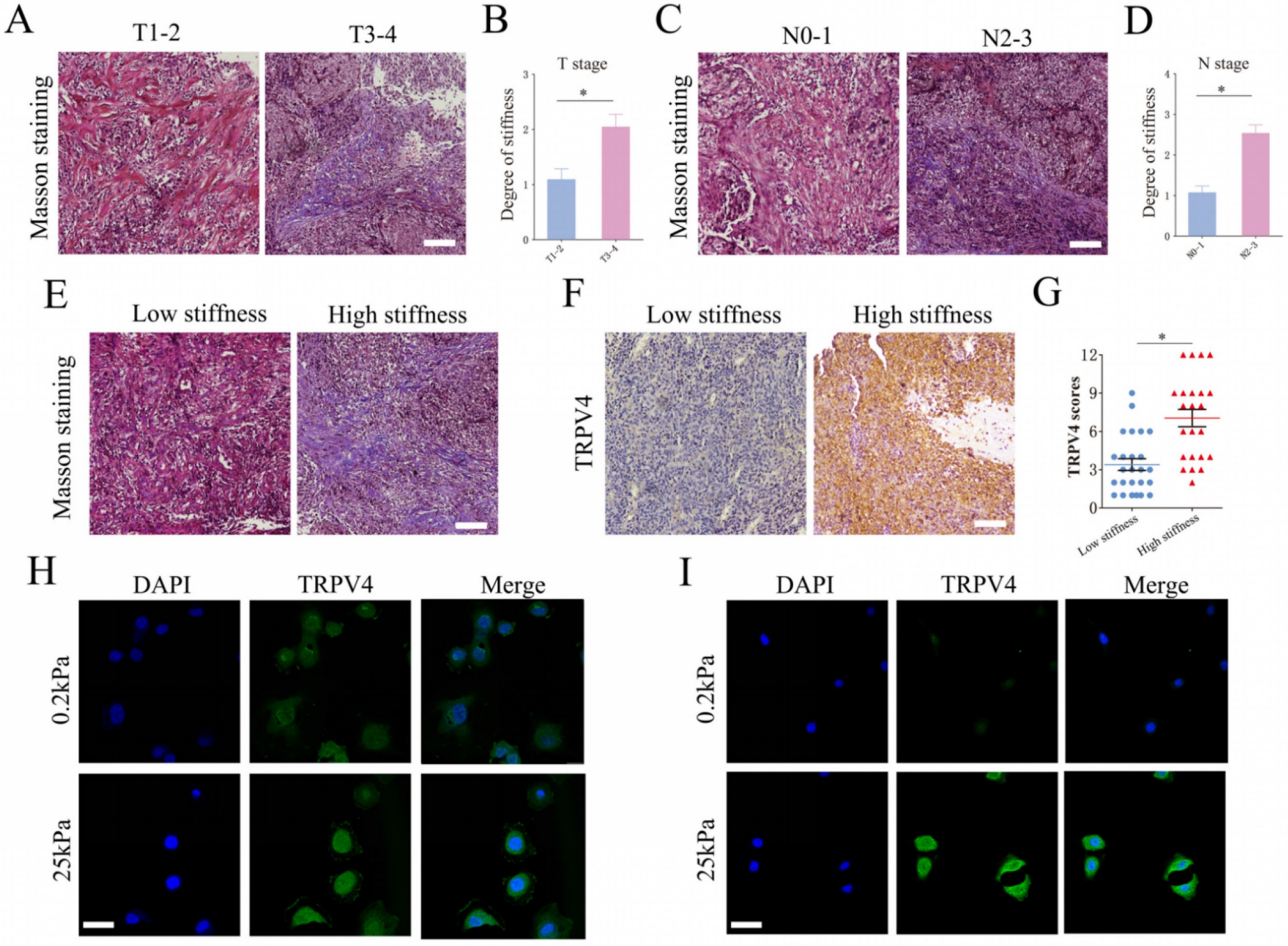
** Matrix stiffness correlates with advanced stages and high TRPV4 expression in NPC tissues.** (A-D) Representative images of Masson's trichrome staining and summary data of matrix stiffness in NPC tissues of different T-stages and N-stages. Scale bar = 100μm. (E) Representative images of low-stiffness and high-stiffness NPC tissues, scale bar = 100μm. (F-G) Representative images and scores of TRPV4 expression in low-stiffness and high-stiffness NPC tissues, scale bar = 100μm. (H-I) Representative immunofluorescence images of TRPV4 in the 5-8F and CNE1 cell lines grown under different stiffness conditions, scale bar = 50μm. *, P < 0.05.

We analyzed the matrix stiffness in NPC tissue specimens by staining for collagen fibers. The T3-4 stage tumors showed an increase in matrix stiffness compared to the tumors of T1-2 stages (Fig. 1A-B). Furthermore, the N2-3 stage tumors had a higher level of matrix stiffness (Fig. 1C-D). Based on the percentage of collagen fibers, the NPC specimens were classified as low-stiffness (score 0 to 2) and high-stiffness (score 3 to 4). As shown in Fig. 1E-G, the high-stiffness tissues expressed higher levels of TRPV4 protein compared to the low-stiffness tissues. To simulate matrix stiffness *in vitro*, we cultured NPC cells at 0.2 kPa (low stiffness) and 25 kPa (high stiffness). TRPV4 expression was significantly higher in cells subjected to 25 kPa compared to the cells cultured at 0.2 kPa (Fig. 1H-I). Taken together, our findings suggest that higher matrix stiffness correlates with advanced clinical stage in NPC, which may increase TRPV4 expression.

### Matrix stiffness promotes invasiveness and EMT of NPC cells

**Figure 2:**
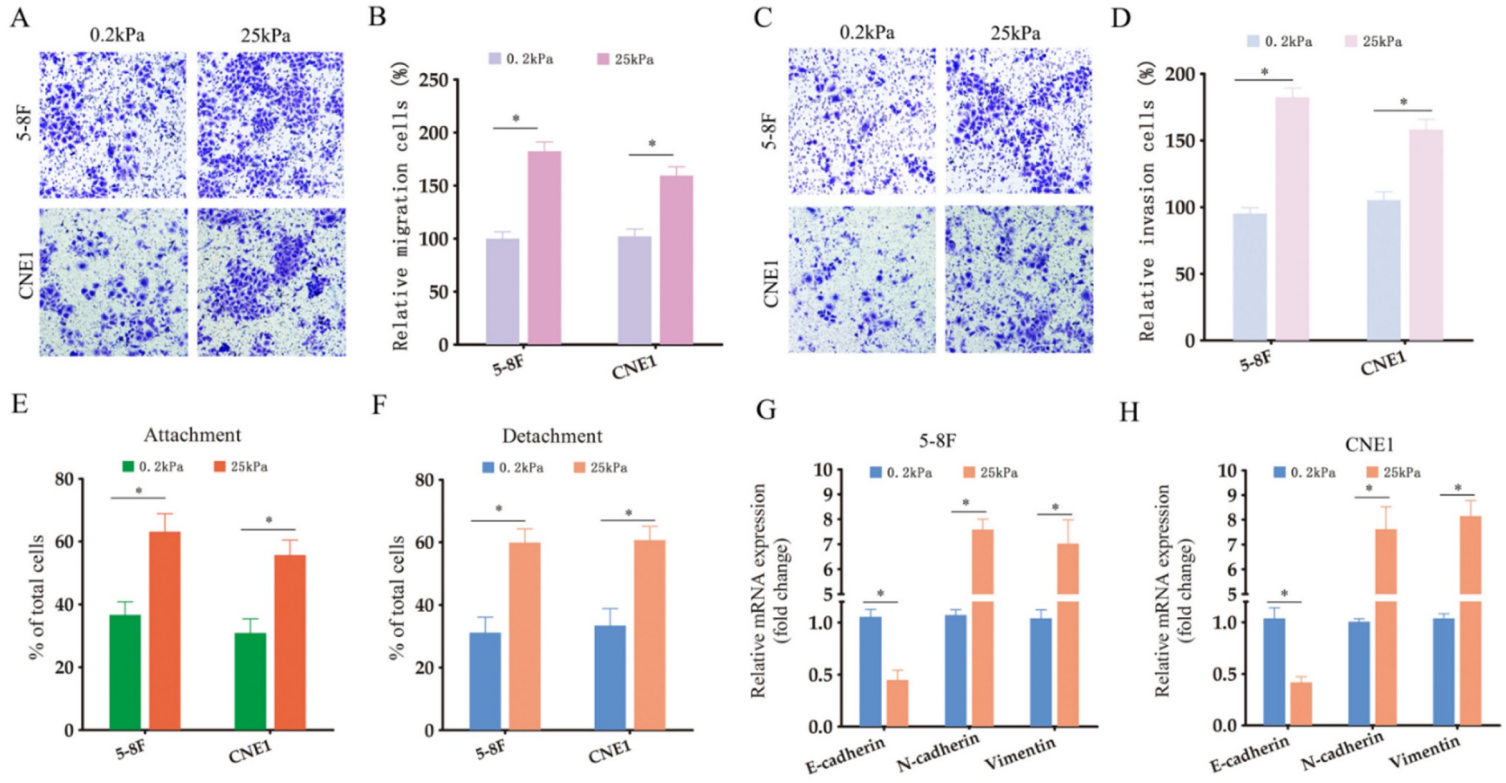
** High stiffness promotes the invasiveness of NPC cells.** (A-B) Representative images and summary data of the migration rates of 5-8F and CNE1 cells under different stiffness conditions. (C-D) Representative images and summary data of the invasive abilities of 5-8F and CNE1 cells under different stiffness conditions. (E-F) Adhesion and detachment abilities of 5-8F and CNE1 cells under different stiffness conditions. (G-H) Expression levels of the indicated EMT markers in 5-8F and CNE1 cells cultured under different stiffness conditions. *, P<0.05, versus 0.2kPa.

In order to explore the role of matrix stiffness in the invasive behavior of NPC cells, we cultured the 5-8F and CNE1 cells under low- and high-stiffness conditions, and analyzed their migration and invasion in transwell assays. The NPC cells grown at 25 kPa exhibited higher migration and invasion rates compared to the cells subjected to 0.2 kPa (Fig. 2A-B). NPC cells cultured on high stiffness surfaces also had stronger adhesion and detachment abilities compared to the low-stiffness counterparts (Fig. 2E-F). To explore the possible regulatory mechanisms underlying matrix stiffness, we examined the expression levels of EMT-related markers.

As shown in Fig. 2G-H, the epithelial marker E-cadherin was downregulated, while the mesenchymal markers N-cadherin and vimentin were upregulated in NPC cell lines under high-stiffness conditions, which is indicative of accelerated EMT. Collectively, our results demonstrated that matrix stiffness can increase the invasiveness and metastatic ability of NPC cells by promoting EMT.

### TRPV4 regulates matrix stiffness-induced invasiveness and EMT in NPC cells

**Figure 3:**
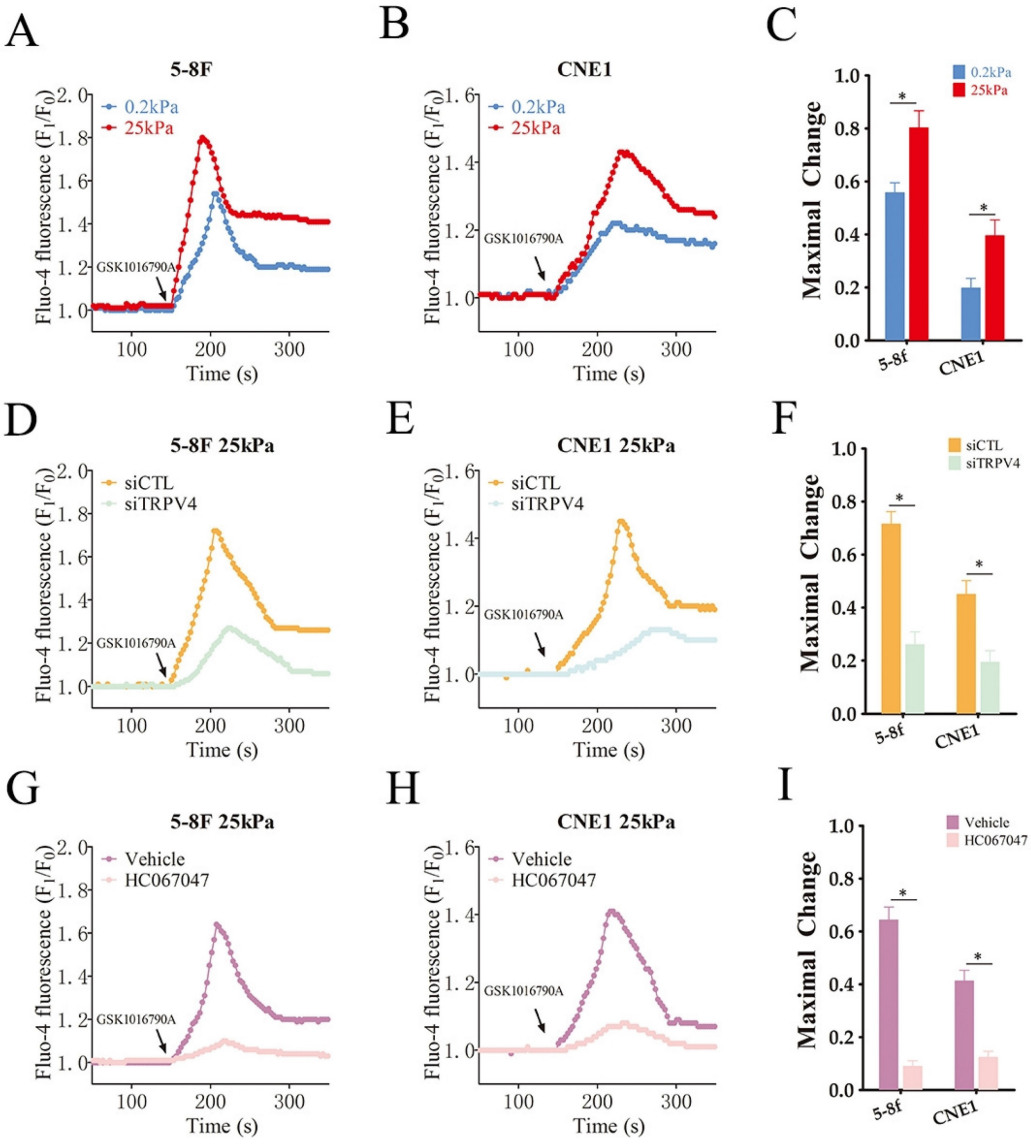
** High matrix stiffness activates TRPV4 function.** (A-C) Representative single measurement of cellular Ca^2+^ in 5-8F and CNE1 cells cultured under low- and high-stiffness conditions with GSK1016790A (100nM). (D-F) Representative single measurements of cytosolic Ca^2+^ in the control (siCTL) and TRPV4-knockdown (siTRPV4) 5-8F and CNE1 cells cultured under high-stiffness conditions with GSK1016790A (100nM). (G-I) Representative single measurements of cytosolic Ca^2+^ in 5-8F and CNE1 cells pre-treated with vehicle (0.1% DMSO) or HC067047 (4μM) under high-stiffness conditions with GSK1016790A. *, P<0.05, versus vehicle or siCTL.

**Figure 4:**
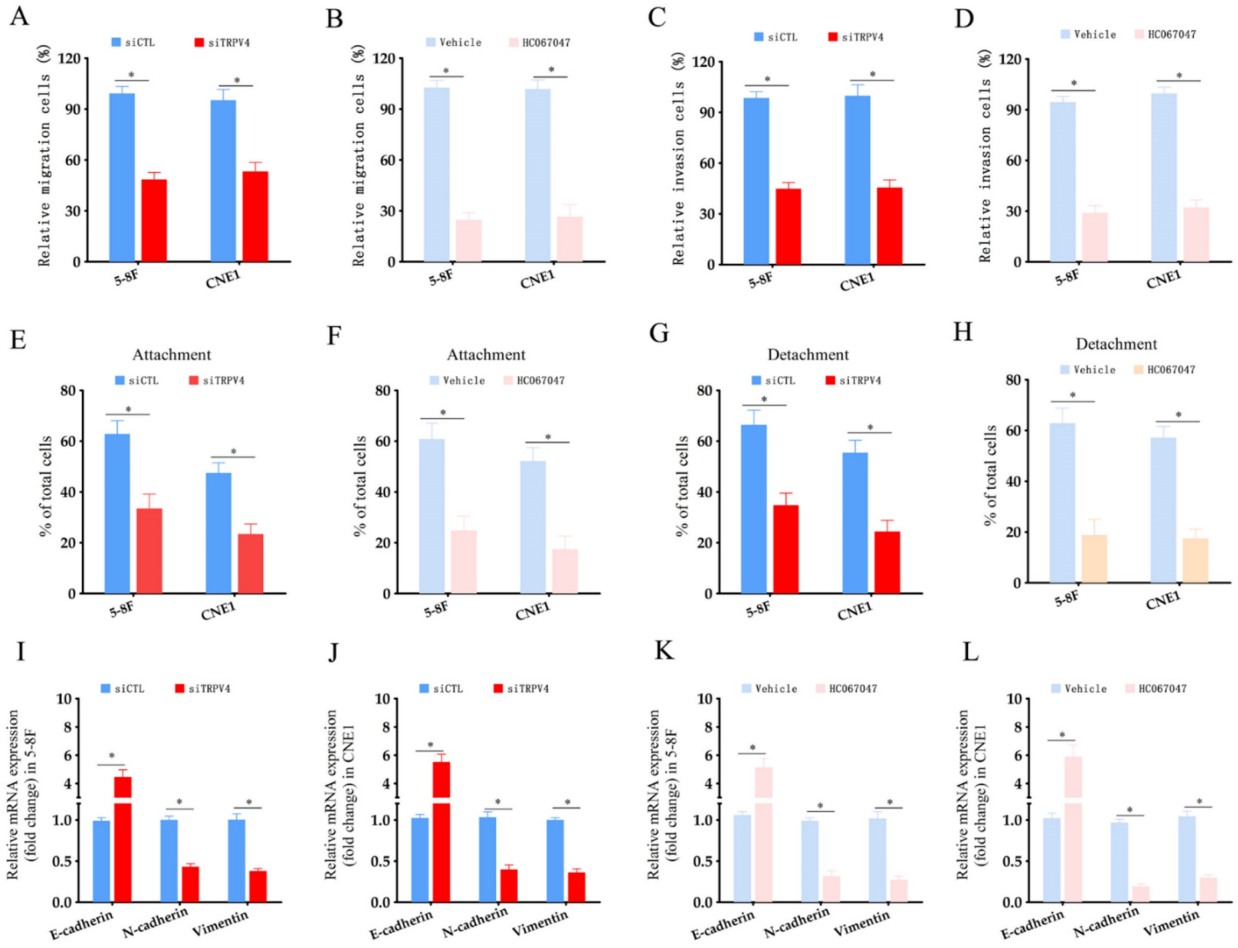
** TRPV4 knockdown decreased the invasiveness of NPC cells under high stiffness conditions.** (A-B) Migration rates of 5-8F and CNE1 cells transfected with siCTL and siTRPV4 or treated with vehicle and HC-067047 under high-stiffness conditions. (C-D) Percentage of invasive 5-8F and CNE1 cells transfected with siCTL and siTRPV4 or treated with vehicle and HC-067047 under high-stiffness conditions. (E-H) Adhesion and detachment of NPC cells treated as in A and B. (I-L) Relative expression levels of EMT markers in A and B. *, p<0.05, versus vehicle or siCTL.

To explore the functional relevance of TRPV4, we measured Ca^2+^ flux in the NPC cells grown under high- and low-stiffness conditions using Fluo-4/AM staining. Following stimulation with the TRPV4 agonist GSK1016790A, the Ca^2+^ influx increased significantly in the high-stiffness condition group compared to the low-stiffness condition group (Fig. 3A-C). On the other hand, TRPV4 knockdown suppressed the Ca^2+^ influx induced by GSK1016790A under high-stiffness conditions (Fig. 3D-F and Supplementary Figure S1). Likewise, pharmacological inhibition of TRPV4 using HC-067047 effectively inhibited GSK1016790A-induced Ca^2+^ influx (Fig. 3G-I). Thus, TRPV4 may exert its biological effects on NPC cells with high matrix stiffness through the Ca^2+^ influx. Both genetic and pharmacological inhibition of TRPV4 significantly reduced the migration and invasion abilities of NPC cells under high-stiffness conditions (Fig. 4A-D), and impaired their adhesion and detachment (Fig. 4E-H). Consistent with this, knockdown or blockade of TRPV4 increased the relative expression of E-cadherin and decreased that of vimentin and N-cadherin (Fig. 4I-L). Taken together, these results suggest that TRPV4 regulates the matrix stiffness-induced invasiveness and EMT of NPC cells.

### The TRPV4/NOX4/IL-8 axis is critical for matrix stiffness-induced invasiveness and EMT in NPC cells

**Figure 5:**
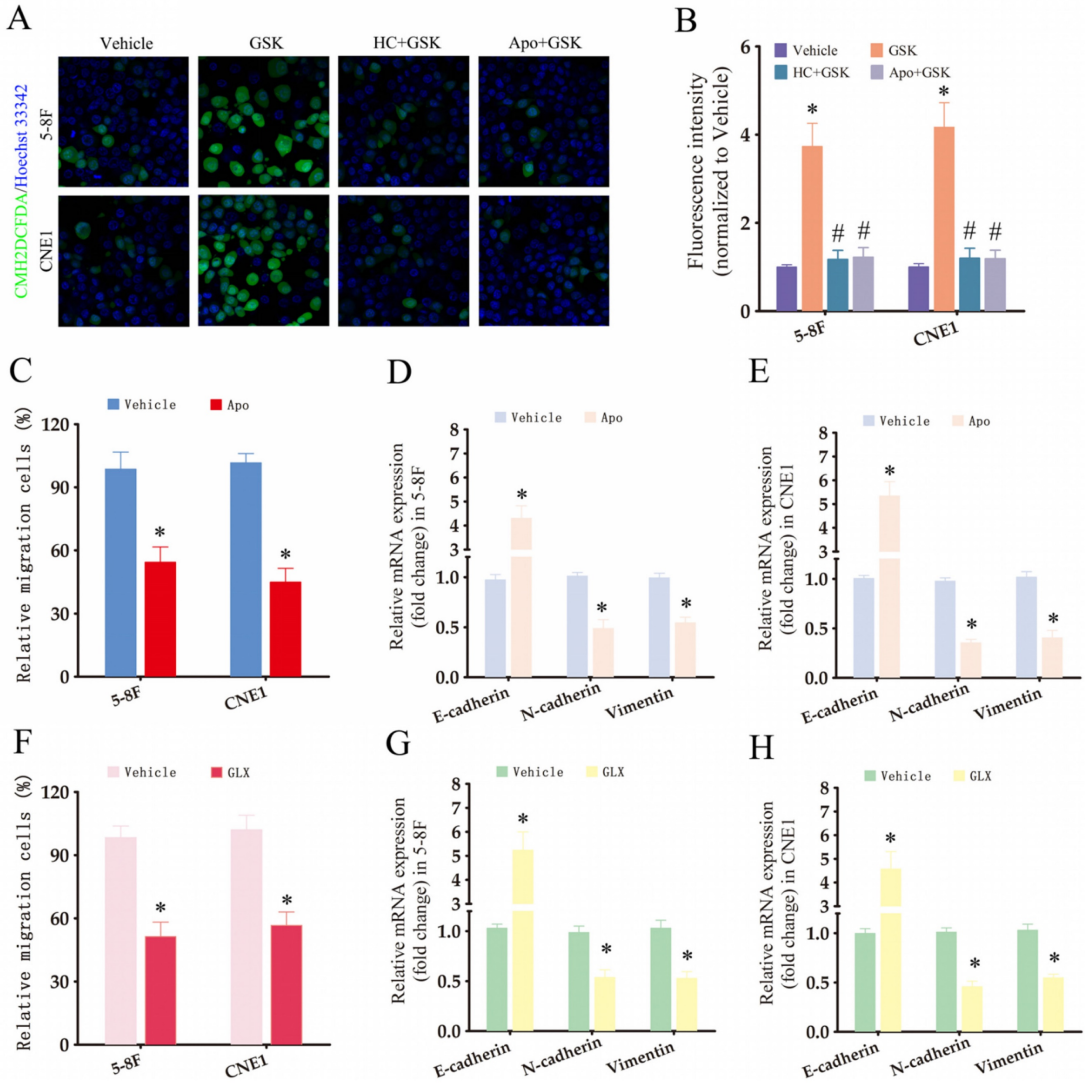
** The TRPV4/NOX4 axis is critical for matrix stiffness-induced invasiveness and EMT in NPC cells.** (A-B) Representative images and summary data of ROS levels in the 5-8F and CNE1 cells treated with vehicle (0.1% DMSO), GSK1016790A (GSK), HC-067047+GSK1016790A (HC+GSK), and apocynin+GSK1016790A (APO+GSK) under high-stiffness conditions. (C-E) Migration rates and expression of EMT markers in 5-8F and CNE1 cells treated with vehicle and apocynin under high-stiffness conditions. (F-H) Migration rates and expression of EMT markers in 5-8F and CNE1 cells treated with vehicle and GLX351322 (GLX) under high-stiffness conditions. *, P<0.05, versus vehicle; #, P<0.05, versus GSK.

To further investigate the downstream effectors of TRPV4, we measured ROS production in the NPC cells using the CM-H2DCFDA probe. Interestingly, TRPV4 activation in NPC cells with high-stiffnesss condition under enhanced ROS production (Fig. 5A-B), while pharmacological inhibition of TRPV4 (HC067047) or NOX (apocynin) suppressed GSK1016790A-induced ROS production (Fig. 5A-B), indicating that TRPV4 may regulate ROS levels through NOX. Apocynin, a broad-spectrum NOX inhibitor, significantly attenuated the migration and EMT of NPC cells grown under high-stiffness conditions (Fig. 5C-E). The NOX4 antagonist GLX351322 showed similar inhibitory effects (Fig. 5F-H). Based on these results, we hypothesized that matrix stiffness in NPC may promote migration and EMT through the TRPV4-NOX4 axis.

**Figure 6:**
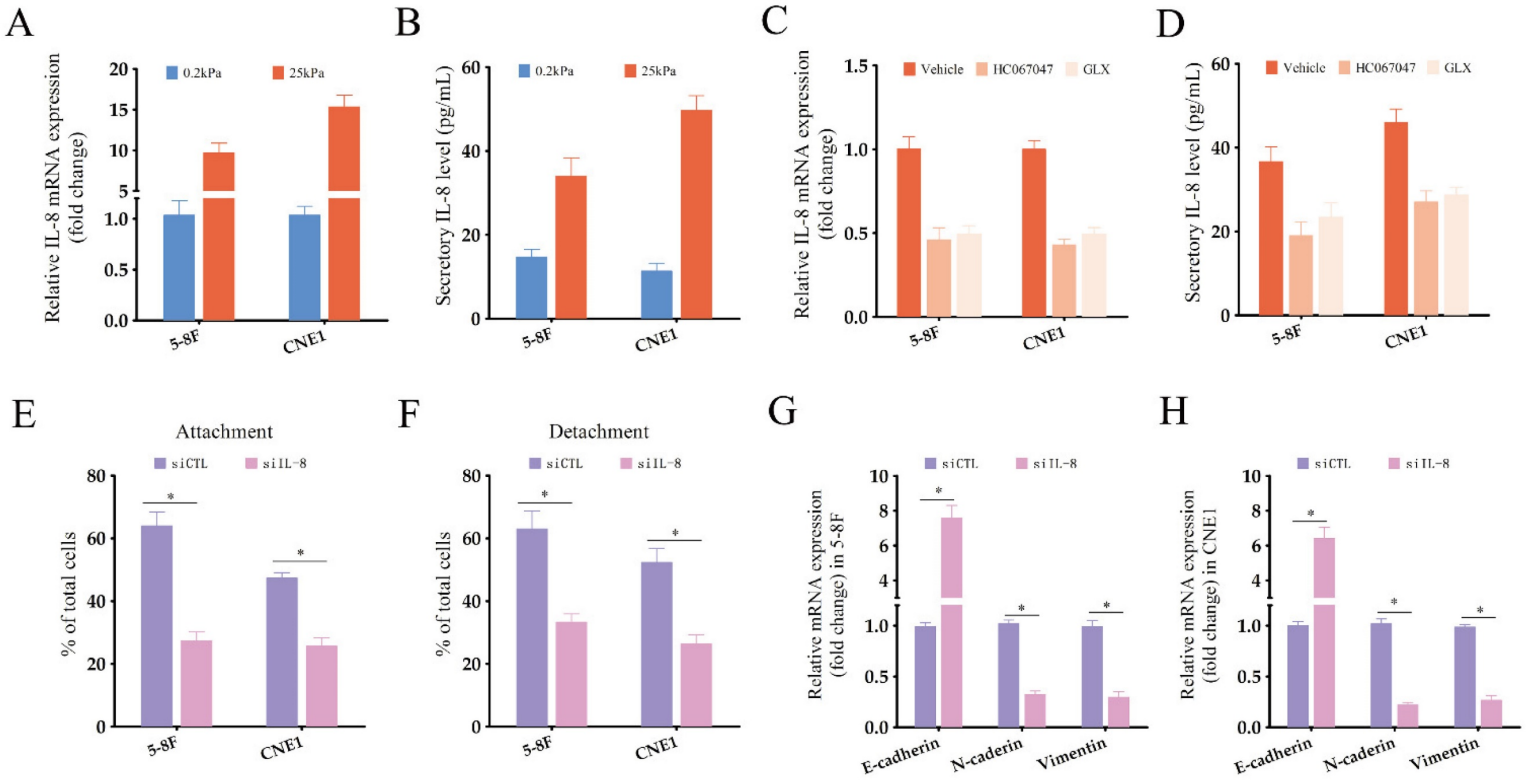
** Matrix stiffness increases IL-8 production in NPC cells.** (A-B) Relative expression of IL-8 mRNA and protein in NPC cells grown under different stiffness conditions. (C-D) Relative expression of IL-8 mRNA and protein in NPC cells treated with Vehicle, HC-067047 and GLX351322 under high-stiffness conditions. (E-F) Percentage of adherent and detached NPC cells transfected with siCTL or siIL-8. (G-H) Relative expression levels of EMT markers in NPC cells treated as in E-F.*, P<0.05, versus 0.2kPa, vehicle or siCTL.

**Figure 7:**
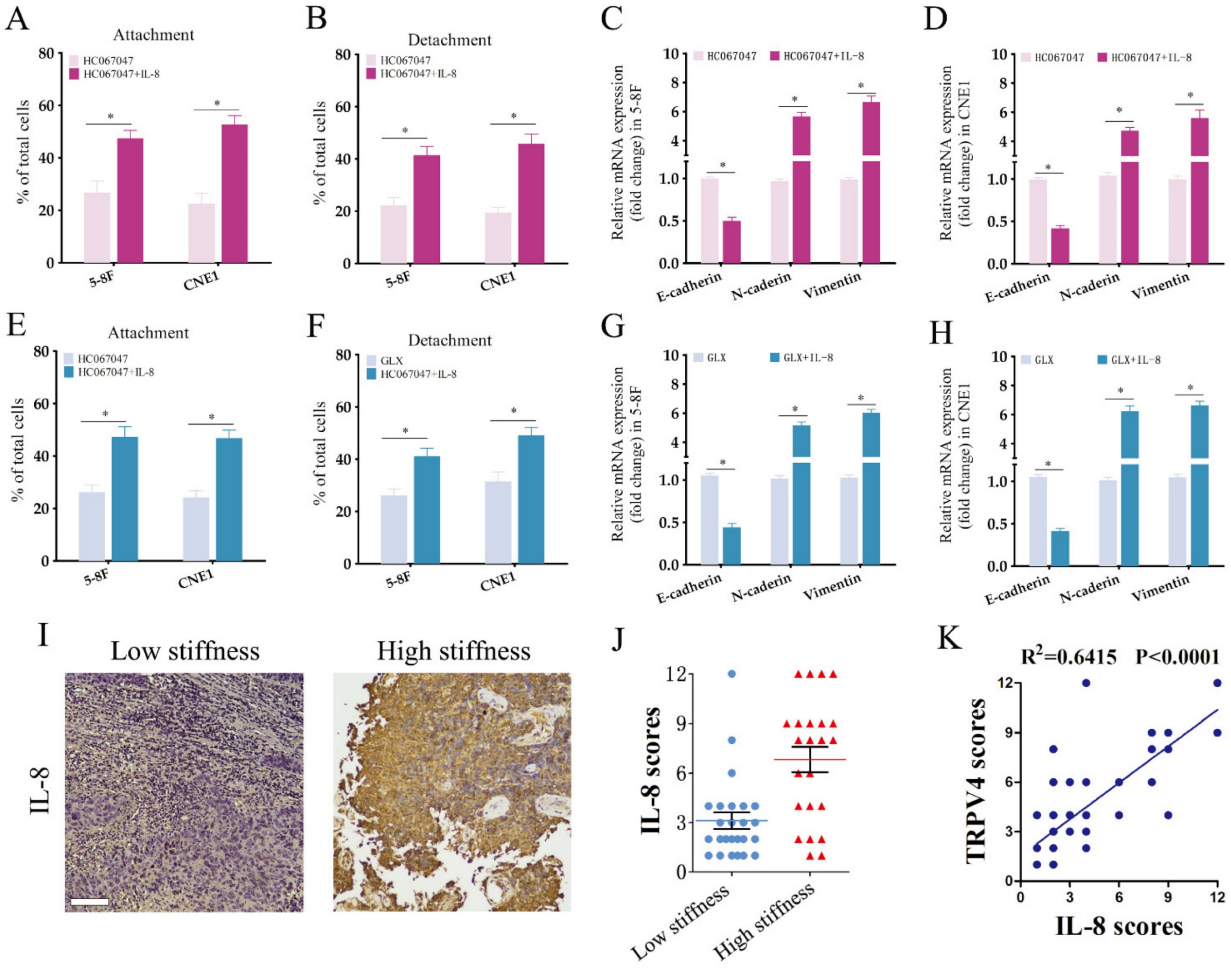
** IL-8 is the target of TPRV4/NOX4 axis in NPC cells.** (A-B) Attachment and detachment of NPC cells treated with HC-067047 and IL-8. (C-D) Relative expression levels of EMT markers in A and B. (E-F) Attachment and detachment of NPC cells treated with GLX351322 (GLX) and IL-8. (G-H) Relative expression levels of EMT markers in E and F. (I-J) Representative images and scores of IL-8 protein expression in low- and high-stiffness NPC tissues. (K) Pearson correlation of IL-8 expression with TRPV4 expression. *, P<0.05, versus HC or GLX.

NPC cells grown under high-stiffness conditions showed significant upregulation of IL-8 mRNA, and also secreted high levels of the cytokine (Fig. 6A-B). Therefore, we next investigated whether the TRPV4/NOX4 axis also regulates IL-8 production in response to matrix stiffness. As shown in Fig. 6C, blocking TRPV4 or Nox4 significantly reduced IL-8 RNA expression and secretion. Furthermore, IL-8 knockdown significantly decreased the adhesion and detachment abilities of the NPC cells (Fig. 6E-F and Supplementary Figure S2), and suppressed EMT (Fig. 6G-H) compared to the control group. To further validate the effector role of IL-8 in the TRPV4/NOX4 axis, we treated NPC cells with the TRPV4 inhibitor HC067047 and recombinant IL-8. As shown in Fig. 7A-B, IL-8 reversed the inhibitory effect of TRPV4 blockade on the adhesion and detachment abilities of NPC cells with high-stiffnesss condition. Consistent with this, pre-treatment with IL-8 also promoted EMT following TRPV4 inhibition (Fig. 7C-D). Likewise, IL-8 also enhanced the adhesion, detachment and EMT of NPC cells under high-stiffnesss condition with NOX4 inhibition (Fig. 7E-H). Finally, immunostaining of NPC tissue specimens showed that the in-situ IL-8 protein expression was significantly increased in the high-stiffness NPC tissues (Fig. 7I-J). In addition, IL-8 expression correlated positively with the expression of TRPV4 (Fig. 7K). Together, our findings suggest that matrix stiffness mediates the invasiveness of NPC cells via the TRPV4/NOX4/IL-8 axis.

## Discussion

The tumor stroma exhibits increased stiffness due to changes in the cell types and ECM 20, which in turn drives cancer progression 21. For instance, the stiffness of breast tumors stimulates the invasion and metastasis of the malignant cells 22. Similarly, tumor stiffness can drive the EMT of prostate cancer cells 23. Consistent with these findings, the stiffness of NPC tissue specimens increased with the progression of tumor stage. Furthermore, the expression levels of TRPV4 were significantly increased in the high-stiffness NPC tissues, as well as in NPC cells cultured at 25 kPa to simulate matrix stiffness. TRPV4 is a non-selective cation channel that is highly sensitive to calcium ions 24, and can be activated by multiple physiological and pathological stimuli, such as temperature, pressure, biochemical stimuli and osmotic pressure. Activation of TRPV4 increases calcium influx, and high intracellular Ca^2+^ levels regulate various biological processes 25. Previous studies have shown that tumor stiffness activates TRPV4 and induces Ca^2+^ influx, which can further alter matrix stiffness by modulating the cytoskeleton and enzyme activities 26. TRPV4 was involved in mediating stiffness-induced foreign body response and giant cell formation 27, 28. TRPV4 regulated breast cancer cell extravasation and stiffness by controlling the cytoskeleton at the cell cortex 29. In our study, TRPV4-driven Ca^2+^ influx was markedly increased in the high-stiffness NPC cells. Pharmacological inhibition or gene-silencing of TRPV4 suppressed the matrix stiffness-induced invasiveness of NPC cells 30. This led us to hypothesize that matrix stiffness regulates invasiveness of NPC cells by mediating Ca^2+^ activity through TRPV4. Consistent with our findings, tumor stiffness promotes the proliferation of oral cancer cells by mediating Ca^2+^ influx through TRPV4 31. Furthermore, TRPV4 regulates the cytoskeleton of endometrial cancer cells through the RhoA/ROCK1 pathway, thereby promoting metastasis 32.

Pre-treatment with TRPV4 agonists increased the production of intracellular ROS in NPC cells grown under high-stiffness conditions. ROS or free radicals are highly active molecules that play a dual role in living organisms 33. Under normal physiological conditions, appropriate levels of ROS mediate various cellular activities and immune responses. However, excessive ROS production causes oxidative damage to tissues, which has been linked to tumorigenesis and progression. For instance, high levels of ROS regulate the EMT and apoptosis of oral cancer cells, thus promoting tumor progression 34. Likewise, aberrant ROS production correlates with therapy resistance of HCC cells 35. We found that inhibiting ROS production in the NPC cells by blocking NOX4 decreased the invasiveness induced by matrix stiffness. In a previous study, we had shown that the TRPV4/NOX2 axis promoted vascular permeability in obese mice by inducing ROS generation 36. Accordingly, we hypothesized that matrix stiffness may regulate the invasiveness and EMT of NPC cells through the TRPV4/NOX4 axis.

IL-8 is a pro-inflammatory CXC chemokine that promotes tumor angiogenesis and metastasis, and is associated with poor prognosis in NPC 37. Previous studies have shown that the production of IL-8 is dependent on ROS generation 38, 39. Therefore, we hypothesized that IL-8 may function downstream of the TRPV4/NOX4 axis. Indeed, NPC cells grown under high-stiffness conditions showed elevated IL-8 mRNA expression and also secreted higher levels of IL-8. Moreover, knocking down IL-8 significantly decreased the adhesion, detachment and EMT of the high-stiffness NPC cells. Additionally, pre-treatment with IL-8 rescued the inhibitory effect of TRPV4/NOX4 blockade on the invasiveness of NPC cells. Both TRPV4 and NOX4 levels were significantly higher in the high-stiffness NPC specimens, and showed a positive correlation. Moreover, IL-8 signaling was reported as one of the most important altered pathways in NPC, our findings may partly explain the unstisfying response to chemo/immunoherapies. Targeting TRPV4/NOX4/IL-8 axis may be a potential strategy for combining chemo/immunoherapies. Nevertheless, future studies are needed to test this hypothesis.

## Conclusion

Matrix stiffness can regulate the invasive behavior of NPC cells through the TRPV4/NOX4/IL-8 signaling axis. Advanced clinical stage of NPC is associated with increased matrix stiffness and overexpression of TRPV4 and IL-8. Our findings suggest that the TRPV4/Nox4/IL-8 axis is a promising target for NPC therapy.

## Supplementary Material

Supplementary figures and tables.

## Funding

Guangdong Basic and Applied Basic Research Foundation (2021A1515010970); Shenzhen Innovation of Science and Technology Commission (No. JCYJ20230807091702005, JCYJ20210324132407019); Longgang Innovation of Science and Technology Commission (LGKCYLWS2022002, LGKCYLWS2021000027); Shenzhen Key Medical Discipline Construction Fund (No. SZXK039); Longgang Medical Discipline Construction Fund (Key Medica Discipline in Longgang District).
