# Supplementary material for: Matrix stiffness regulates NPC invasiveness by modulating a mechanoresponsive TRPV4-Nox4-IL-8 signaling axis
Source: J Cancer. 2025 Jan 13;16(4):1324–34. doi: 10.7150/jca.104235 (PMC11786026; doi:10.7150/jca.104235)
Supplement: Supplementary file 1 — Supplementary figures and tables. [file jcav16p1324s1.pdf]

Supplementary information

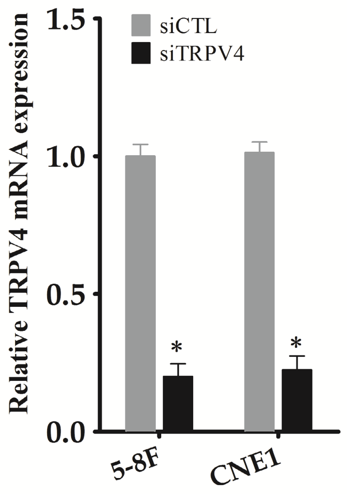

Figure S1. TRPV4 mRNA levels in 5-8F and CNE1 cells transfected with siRNA against TRPV4. Values are means ± SEM, n=4 *, p<0.05 compared to siCTL.

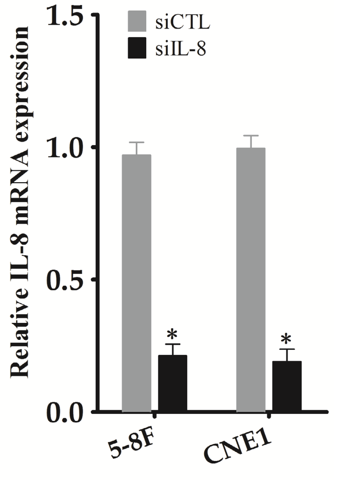

Figure S2. IL-8 mRNA levels in 5-8F and CNE1 cells transfected with siRNA against IL-8. Values are means ± SEM, n=4 *, p<0.05 compared to siCTL.

Table S1 Primer sequences used for Real-time PCR

Gene Name	Primer	Sequence
E-cadherin	Forward	GCCTCCTGAAAAGAGAGTGGAAG
	Reverse	TGGCAGTGTCTCTCCAAATCCG
N-cadherin	Forward	CCTCCAGAGTTTACTGCCATGAC
	Reverse	GTAGGATCTCCGCCACTGATTC
Vimentin	Forward	AGGCAAAGCAGGAGTCCACTGA
	Reverse	ATCTGGCGTTCCAGGGACTCAT
TRPV4	Forward	TACGAGAGTGCTCGCATCCTCA
	Reverse	TGTCTTCAGGCTACATGAGCCG
IL-8	Forward	AGACAGCCACTCACCTCTTCAG
	Reverse	TTCTGCCAGTGCCTCTTTGCTG
Actin	Forward	CACCATTGGCAATGAGCGGTTC
	Reverse	AGGTCTTTGCGGATGTCCACGT

Table S2 Target sequences used for silencing

Gene Name	Target sequence
TRPV4	siRNA:5’- GCCGTCTCCTTCTACATCAACGTGGTCTC -3’
IL-8	siRNA:5’- CCGAACTTTAATTTCAGGAAT -3’
Control	siRNA:5’- GCTTCGCGCCGTAGTCTTATCA -3’
